# The Relationship between Same-Day Access and Continuity in Primary Care and Emergency Department Visits

**DOI:** 10.1371/journal.pone.0135274

**Published:** 2015-09-02

**Authors:** Jean Yoon, Kristina M. Cordasco, Adam Chow, Lisa V. Rubenstein

**Affiliations:** 1 Health Economics Resource Center, VA Palo Alto, Menlo Park, CA, United States of America; 2 Center for Innovation to Implementation, VA Palo Alto, Menlo Park, CA, United States of America; 3 Center for the Study of Healthcare Innovation, Implementation, & Policy, Greater Los Angeles VA, Sepulveda, CA, United States of America; 4 UCLA School of Medicine, Los Angeles, CA, United States of America; 5 RAND Corp, Santa Monica, CA, United States of America; University of California, San Francisco, UNITED STATES

## Abstract

We examined how emergency department (ED) visits for potentially preventable, mental health, and other diagnoses were related to same-day access and provider continuity in primary care using administrative data from 71,296 patients in 22 VHA clinics over a three-year period. ED visits were categorized as non-emergent; primary care treatable; preventable; not preventable; or mental health-related. We conducted multi-level regression models adjusted for patient and clinic factors. More same-day access significantly predicted fewer non-emergent and primary care treatable ED visits while continuity was not significantly related to any type of ED visit. Neither measure was related to ED visits for mental health problems.

## Introduction

Many health care systems are investing in new models of care such as accountable care organizations and patient-centered medical homes in order to strengthen access, continuity of care, and care coordination while promoting quality and safety. These new models frequently target the reduction of emergency department (ED) care since reducing potentially preventable or unnecessary ED visits may improve efficiency of care and patient outcomes overall. There is limited evidence regarding the ability of core features of primary care, such as timely access to care and provider continuity, to impact ED visits, especially for diagnoses that are treatable in primary care. Previous research found that poor access as measured by lacking a usual source of care, longer waiting times for appointments, and less provider availability and worse continuity with a primary care provider were associated with having ED visits [1–3]. In addition, many health care organizations have attempted to improve access and continuity within patient-centered medical home (PCMH) models and have found this model of care reduced ED visits [4–8], so improving primary care systems may contribute to preventing unnecessary ED use.

ED visits for mental health, in particular, are a growing burden on emergency departments [9–11], yet it is unknown to what extent attributes of primary care can influence use of ED care for mental health. ED visits for mental health conditions may be less responsive to primary care access and continuity than conditions more easily treated in primary care.

The Veterans Health Administration’s (VHA) system cares for approximately 8.3 million patients in integrated care networks including primary, secondary, and tertiary care. The VHA system has undergone a series of reorganizations and transformations of its delivery system beginning in 1996 to emphasize quality improvement and primary care for all patients [12]. Recent initiatives include primary care-mental health integration (PC-MHI) which began in 2007 to disseminate nationally the use of collaborative mental health resources in primary care practices, and in 2010 the national implementation of PCMH in all VHA primary care clinics, which was called Patient Aligned Care Teams (PACT) to emphasize the team-based approach. In concert with achieving team-based care, there has been a focus on improving access, provider continuity, care management, and coordination [13]. VHA also adopted several measures to track medical home implementation progress including measures of same-day access and provider continuity. It is unknown if these features of primary care are related to ED visits.

In this study we seek to assess the relationship between key features of primary care—same-day access and provider continuity—and different types of ED visits in 22 VHA primary care clinics. We used differences over time and between clinics in same-day access and care continuity measures to determine the associations with ED visits. We hypothesized that higher same-day access and provider continuity were associated with less ED care for conditions not requiring emergency care or treatable in primary care but would have less impact on emergency care for non-preventable and mental health conditions.

## Methods

### Study Cohort and Data Sources

The study included 22 primary care clinics in three VHA medical systems in Southern California. We identified patients who visited these clinics at least twice during fiscal year (FY) 2009 (October 1, 2008 to September 30, 2009). We excluded patients with less frequent use of primary care and those who died between 2009 and 2012.

We aggregated utilization, diagnoses, and patient characteristics for the cohort for a 3-year period following PCMH implementation from FY2010 through FY2012. Patients were assigned a “home clinic” where they received a plurality of primary care visits and linked to clinic administrative data including clinic PCMH performance measures. We obtained patients’ utilization from VHA administrative records that were not individually identifiable, so patients’ consent to use their data was not obtained. This study received approval from the Stanford University IRB (protocol #20124).

### Measures

Dependent Variables: We estimated the total number of ED visits per patient by type in each study year (FY2010-FY2012). ED visits were obtained from outpatient records from VHA facilities and non-VHA facilities with VHA payment. Using a previously validated algorithm,[14–16] we obtained the primary diagnosis for each ED visit and assigned the visit a probability of being in the following categories: 1. non-emergent (care was not required within 12 hours, e.g. urinary tract infection); 2. emergent and primary care treatable (treatment required within 12 hours, but care could have been provided in primary care setting, e.g. CAT scan, certain lab tests); 3. ED care needed but preventable (condition potentially preventable if timely ambulatory care was provided, e.g. asthma exacerbation, hyperglycemia); 4. ED care needed and not preventable (condition could not have been prevented with ambulatory care, e.g. trauma, appendicitis); 5. psychiatric condition; 6. alcohol use; and 7. drug use [14–16]. We also grouped psychiatric condition, alcohol use, and drug use under mental health for adjusted analyses because these visits were fewer in number. ED visits assigned as emergent under this algorithm have been associated with higher odds of hospitalization and mortality.[14]

We estimated the total number, or fraction, of ED visits by category for each patient in each year. For example, if a patient had one ED visit in a study year that was 50% likely of being non-emergent and 50% likely of being emergent and primary care treatable, his total number of ED visits would be counted as 0.5 non-emergent and 0.5 emergent and primary care treatable visits for that year.

Main Independent Variables: Our main independent variables were clinic-level measures of access and provider continuity FY2010-FY2012. Beginning in FY2010 VHA established national clinic-level measures, including annual measures of access and continuity, to track PCMH progress. We measured same-day access as the percent of all patients receiving a primary care appointment within 1 day of a same-day requested appointment in that clinic. We measured provider continuity as the average percent of all outpatient visits with patients’ assigned primary care provider (PCP) for patients in the clinic.

Other Independent Variables: We measured several patient-level independent variables including health status that can increase ED use. The Charlson Index using the Deyo-Quan approach [17] was measured for each patient and categorized into groups of low and high scores. The Charlson Index was developed to predict 10-year mortality based on a range of comorbid conditions [18]. For descriptive purposes limited to bivariate analyses, we also measured presence of several chronic conditions to see if specific conditions were associated with ED use. We measured patient diagnoses based on ICD-9 codes recorded in encounters for the indicated year. To avoid counting diagnoses recorded to rule out conditions, we required at least two separate encounters coded with a diagnosis to indicate that a patient had the diagnosis. We assessed presence of these conditions: asthma, chronic heart failure, chronic obstructive pulmonary disease, diabetes, hypertension, ischemic heart disease, depression, psychiatric disorders (including post-traumatic stress disorder, antisocial personality disorder, schizophrenia, borderline disorder, manic depression, and other psychiatric disorders), and alcohol or drug use disorders. Another measure of health status or need was represented by counts of the total number of primary care visits and telephone encounters.

We also measured patient factors predominantly fixed over time from the year the cohort was drawn (FY2009) since low socioeconomic status, lack of housing, greater disability, and poor social support were hypothesized to be associated with more ED utilization. These characteristics included age, gender, race/ethnicity, marital status, means test, homelessness, and service-connected disability. Patients’ eligibility for VHA care was categorized according to eligibility rules as either 1. having a service-connected disability or those recently discharged, 2. at or below VA’s income limits under a means test, or 3. above VA’s income limits under a means test. Patients were also grouped based on the degree their disability was service-connected (0%, 1–50%, 51–100%).

Several other clinic-level measures were also included because larger clinics with sicker patients and those closest to a VHA ED may have more VHA ED use. Clinic rurality was assessed through metropolitan/non-metropolitan codes from the Area Resource File (ARF) [19]. Clinic type was categorized as VHA medical center (VAMC) based or community-based outpatient clinic (CBOC). Distance from each clinic to its parent VA medical center was estimated using driving distance calculated by Google maps between the street addresses of the clinics and VA medical centers obtained from the VHA Planning Systems Support Group.

### Analysis

We examined time trends in mean number of ED visits per patient by type of ED visit and mean clinic access and continuity measures and compared them across study years using one way ANOVA. Bivariate analyses were used to compare the mean annual number of ED visits of any type across study years combined to determine how they varied by patient and clinic factors using one way ANOVA. Additional information on standard deviations for the estimates in tables are presented in S1 Table and S2 Table.

We conducted multivariate analyses with count data models since many patients (76%) did not have any ED visits during each study year, and a few patients had more than one. Tests indicated over-dispersion in the distribution of ED visits, so we used negative binomial models because they are appropriate in such cases. Negative binomial models can be used when outcomes are estimated (and not strictly integers) [20].

We conducted six separate regressions for all types and specific types of ED visits. Regression models adjusted for year, patient sociodemographic characteristics (age, gender, race/ethnicity, marital status, means test, service connection, homelessness), patient health status characteristics (Charlson Index, number of annual primary care visits, number of annual telephone visits), and clinic factors (access, continuity, type, metropolitan area, distance to VA medical center).

Both patient-level and clinic-level variables were included in our regressions, so hierarchical models with patient random effects were employed. Random effects models were used since there were multiple observations per patient, and these observations were assumed to be not independent. Patient-specific random effects are assumed to be uncorrelated with the independent variables. Additionally, standard errors were adjusted for the correlation between patients in a clinic [21]. We report the incidence rate ratios which represent the ratio of incidence rates of ED visits for a unit change in a continuous independent variable or change in value for a dummy variable. A significance level of P<0.01 level was used because of the multiple outcomes being tested. We conducted all data analyses in Stata 13.0.

## Results

There were 71,296 primary care VHA patients in the study cohort. Among these patients, there was a rate of 58.1 all-cause ED visits per 100 primary care patients in 2012, a slight increase from 55.5 visits per 100 patients in 2010 (P = 0.0117) (Table 1). There were 11.3 non-emergent ED visits per 100 patients in 2012, which was a slight increase over the study period (P<0.0001). There were 11.8 emergent but primary care treatable ED visits in 2012, with no significant change over time (P = 0.2666). There were 4.3 ED visits per 100 patients for ED care needed but preventable conditions and 8.0 ED visits per 100 patients for ED care needed and not preventable conditions in 2012. ED visits for mental health diagnoses (3.1 per 100 patients in 2012) represented a small portion of total ED visits, and these ED visits were mostly for psychiatric conditions. Psychiatric conditions were the only type of mental health visit with a small decrease over the three-year period (P = 0.0002).

**Table 1:** Number of Emergency Department Visits Per 100 Primary Care Patients by Year and Type, N = 71,296.

Type of ED visit	2010	2011	2012	P-value from one way ANOVA
All-cause†	55.5	55.7	58.1	0.0117
Non-emergent	10.2	10.6	11.3	<0.0001
Emergent				
Primary care treatable	11.8	11.4	11.8	0.2666
ED care needed and preventable	4.1	4.4	4.3	0.0355
ED care needed and not preventable	7.6	7.4	8.0	0.0149
All mental health diagnoses	3.5	3.4	3.1	0.0842
Psychiatric	2.2	2.1	1.7	0.0002
Alcohol use	1.1	1.2	1.2	0.6710
Drug use	0.2	0.2	0.2	0.5779

† All-cause ED visits included ED visits that were not categorized into one of the specific categories listed here.

Over the three-year period following medical home implementation, study clinics had higher mean measures of same-day access and provider continuity, but these differences were not statistically significant (Table 2). Among the 22 primary care clinics, 60% of patients (SD = 23), on average, had a reported appointment within one day of a same-day requested appointment in 2012, and this percent was not significantly different across the three years (P = 0.4069). Between clinics, the mean percent of patients with same-day access ranged from 17% to 99% of patients. The mean overall percent continuity with a patient’s PCP was 79% in 2012 (SD = 9), and there was no significant difference across years (P = 0.5722). Continuity varied between clinics from 46% to 95%.

**Table 2:** Primary Care Clinic Access and Continuity by Year, N = 22.

Clinic Measures	2010	2011	2012	
	Mean (SD)	Mean (SD)	Mean (SD)	P-value from one way ANOVA
**Same-day Access**				
Percent of patients receiving same day appointment within 1 day	50 (25)	53 (24)	60 (23)	0.4069
**Provider Continuity**				
Percent of outpatient visits to primary care provider	77 (13)	75 (14)	79 (9)	0.5722

While clinic access and continuity measures did not vary over time, the variation between clinics was significantly associated with all-cause ED visit rates. Bivariate comparisons across all study years showed that in clinics with low same-day access (less than 40% of patients received an appointment within one day of same-day requested appointment), there was a significantly higher ED visit rate than in clinics with higher same-day access (P<0.001) (Table 3). In clinics that reported at or above the average continuity measure (79% or more of outpatient visits with a patient’s PCP), the rate of ED visits was significantly lower compared to clinics with a lower rate of continuity. Clinics that were medical center-based, located in metropolitan areas, or less than 16 miles from the parent VA medical center also had significantly higher ED visit rates (all P<0.001).

**Table 3:** Annual Rate of ED Visits by Patient and Clinic Characteristics FY2010-2012, N = 71,296.

Patient and Clinic Factors	N (%)	Mean ED Visits/100 Patients per Year*	P-value from one way ANOVA
***Clinic factors***	** **	** **	** **
**Access: Percent of patients receiving same day appointment within 1 day**	** **		<0.001
<40%	24530 (34)	60.9	
40%+	46766 (66)	54.1	
**Provider Continuity: Ratio of outpatient visits to primary care provider**	** **		<0.001
<79%	49095 (67)	66.7	
79%+	23993 (33)	34.2	
**Clinic type**	** **		<0.001
VHA medical center-based clinic	20563 (28)	81.4	
Community-based outpatient clinic	52525 (72)	46.3	
**Metropolitan area**	** **		<0.001
No	1925 (3)	25.0	
Yes	71163 (97)	57.3	
**Distance to VHA medical center**	** **		<0.001
<16 miles	40122 (55)	71.2	
16+	32966 (45)	37.5	
***Patient factors***	** **	** **	** **
**Age group**	** **		<0.001
<45	8982 (13)	65.0	
45–54	10414 (15)	74.4	
55–64	24122 (33)	65.2	
65+	27778 (39)	39.4	
**Gender**	** **		<0.001
Female	3920 (5)	75.2	
Male	67376 (95)	55.3	
**Race/ Ethnicity**	** **		<0.001
White	34953 (49)	63.6	
Black	10239 (14)	77.8	
Hispanic	9016 (13)	61.5	
Other/Unknown	17088 (24)	25.9	
**Marital Status**	** **		<0.001
Married	32995 (47)	40.8	
Separated/Divorced/Widowed	25372 (36)	70.7	
Single Never married	12657(18)	69.0	
Unknown	272 (<1)	34.4	
**Homeless**			<0.001
Yes	3951 (5)	143.6	
No	67345 (95)	51.3	
**Means Test**	** **		<0.001
Below Means Test, Not Service Connected	25107 (35)	70.2	
Service Connected	30904 (43)	58.1	
Above Means Test	11103 (16)	26.4	
Other Eligibility	4182 (6)	40.9	
**Service Connected Percent**	** **		<0.001
0	41894 (59)	55.0	
1–50	16121 (23)	50.0	
51–100	13281 (18)	68.9	
**Charlson Index**	** **		<0.001
0	34706 (49)	37.2	
1+	36590 (51)	74.7	
**Chronic conditions**	** **		<0.001
CHF	2890 (4)	155.5	
COPD	6007 (8)	115.8	
Asthma	3101 (4)	113.5	
Ischemic heart disease	10382 (15)	86.7	
Diabetes	19894 (28)	69.5	
Hypertension	42285 (59)	64.8	
Drug use	3440 (5)	197.7	
Alcohol	4533 (6)	156.1	
Depression	15085 (21)	100.4	
Other Psychiatric disorders	22955 (32)	91.0	
PTSD	10454 (15)	88.1	
**Number of primary care visits**	** **		<0.001
0–2	32832 (46)	22.7	
3+	38464 (54)	85.2	
**Number of telephone visits**	** **		<0.001
0	51820 (73)	37.6	
1+	19476 (27)	106.4	

*Annual rate of ED visits per 100 patients is the mean number of ED visits per patient per year across all study years multiplied by 100.

The rate of all-cause ED visits also varied significantly by various patient factors in bivariate comparisons (Table 3). ED visit rates were higher among patients aged 45–54 years, female, and black. Patients who were not currently married, homeless, below the VHA means test, or had a higher service-connected disability rating also reported higher ED rates. Patients with higher Charlson Index scores or diagnosed with conditions such as alcohol and drug use disorders and heart failure also had significantly higher ED rates. Patients with three or more primary care visits during the year had more ED visits compared to patients with less frequent primary care. Patients receiving any telephone care also had significantly higher ED rates compared to those with none. For all comparisons noted, the ANOVA F-test of differences between groups had P<0.001.

In multivariate analyses adjusting for patient sociodemographic factors, health status measures, clinic characteristics, and patient random effects, clinic access remained a significant predictor of ED visits for any cause (Table 4). A 10% increase in patients receiving primary care appointments within one day of requested date was associated with a 6% reduction in all-cause ED visits (IRR = 0.94; P = 0.002). An increase in continuity of 10% among patients in a clinic had no significant association with ED visits (IRR = 1.10; P = 0.012).

**Table 4:** Incidence Rate Ratios of ED Visits by Type from Adjusted Models FY2010-2012, N = 71,296

Clinic Measures	Any ED visit	Non-emergent ED visits	Primary care treatable ED visits	ED care needed, preventable ED visits	ED care needed, not preventable ED visits	All mental health ED visits
**Same-Day Access**						
Percent of patients (in 10s) receiving same day appointment within 1 day	0.94*	0.93*	0.95*	0.96	0.97	1.04
**Provider Continuity**						
Percent (in 10s) of total outpatient visits with PCP	1.10	1.05	1.08	1.06	1.04	1.03

*P<0.01

Incidence rate ratios were obtained from six separate negative binomial regression models predicting annual number of ED visits for each type separately. All regression models adjusted for year, patient factors (age, gender, race/ethnicity, marital status, service connection, means test, homelessness, Charlson Index, number of annual primary care visits, number of annual telephone visits), and clinic factors (clinic type, metropolitan area, distance to VA medical center) in addition to clinic access and continuity measures. All models included a random effect for each patient to account for multiple observations per patient, and standard errors were adjusted for clustering within clinic.

There were also significant associations between access and specific types of ED visits. A 10 point higher percent of patients getting timely access was estimated to reduce non-emergent visits by 7% (IRR = 0.93; P<0.001). For ED visits that were emergent but considered primary care treatable, higher access was also associated with a lower ED visit incidence rate (IRR = 0.95; P<0.001). There were no significant associations between continuity and these types of ED visits.

There were smaller and non-significant associations with both higher access and continuity for ED visits where ED care was needed and care was preventable (P = 0.016 and P = 0.229, respectively), ED visits where ED care was needed and care was not preventable (P = 0.014 and P = 0.257, respectively), and ED visits for mental health conditions including psychiatric, alcohol, and drug use disorders (P = 0.025 and P = 0.335, respectively).

Several patient and other clinic factors were also independently related to ED visits in adjusted models (data not shown). VHA patients had higher all-cause ED visit rates if they were not currently married, homeless, below the means test, treated in medical-center based clinics, in urban areas, had a higher Charlson score, and used more primary care and telephone care (all P<0.01).

## Discussion

We found that same-day access in primary care was significantly related to ED visits. Same-day access, as measured by the reported ability to receive a primary care appointment within one day when a same-day appointment was requested, was significantly associated with fewer ED visits for all-cause, non-emergent care, and care that was primary care treatable. Our provider continuity measure was not significantly related to these types of ED visits, and neither access nor continuity measures were related to other ED visits that were preventable or not preventable and those related to mental health problems. However, our study occurred during early implementation of the patient-centered medical home, so it is possible that after greater experience with PCMH implementation, the effects of access and continuity on different types of ED visits may change as practices continue to improve their interventions.

Since better same-day access and continuity did not appear to affect ED utilization for mental health problems, primary care improvements alone may not be sufficient to provide high-quality ambulatory care to patients with mental illness. Greater access to specialty mental health providers may also play an important role. Collaborative care management is one approach shown to be successful in improving outcomes for patients with depression and may help lower ED visits and other acute care [22–24]. Additional work is needed to help determine what approaches in primary care can be utilized to reduce reliance on ED care for patients with mental health conditions.

Our results are consistent with earlier work in VHA and non-VHA practices using alternate measures of access. A previous study of VHA PCPs found a modest relationship between less access, as measured by higher appointment booking density, and more ED visits [3]. Other non-VA studies have shown that patients with a usual source of care report that more barriers to timely access such as having difficulty scheduling appointments, ability to contact providers after hours, longer waiting times, and limited clinic hours increased the risk of having an ED visit [2,25,26]. However, our work had different results regarding continuity from another VA study that found that patients with higher continuity with their assigned primary care provider had a lower adjusted rate of ED visits.[27] Chaiyachati et al. did not include measures of access and although the practices in their study did not implement open access during the study period, there may have been a wide range in access between providers and practices that were also associated with continuity measures. Another study of Medicaid patients in Delaware that found that continuity of care with one provider decreased the risk of more ED visits [1]. In an integrated health care system such as VHA, access may be a more important factor in reducing ED visits than continuity. Continuity may be more predictive of other aspects of primary care quality.

Our results also suggest that beyond clinic factors, attention to patient factors is also imperative in reducing unnecessary ED visits. The higher rates of ED visits among patients with heart failure and high use of VHA primary care services, in addition to mental illness, that we observed in our cohort was supported by findings from a recent national study of ED visits among all VHA patients including those not in primary care [28]. Our findings were similar to Doran et al. since patients who were homeless, below the means test or not currently married experienced higher ED rates. Therefore, income, housing, and social support appear to strongly influence patients’ reliance on ED care. Interventions to reduce unnecessary acute care should be designed with these needs in mind; for example, case management and housing programs have reduced ED visits for homeless patients [29].

### Limitations

There were several limitations to our study. We obtained data on ED visits provided by VHA providers and non-VHA providers paid by VHA for services, but we did not have information on ED visits from non-VHA providers and covered by other payers. Since we can only measure ED visits covered by VHA, there may have been changes in non-VHA ED visits during the study period that we did not capture. Another limitation is that the 22 primary care clinics in the study that may not be generalizable to VHA primary care clinics nationally since they were limited to Southern California, but they represent diverse sizes and case mix and include both medical center-based and community-based clinics.

Another limitation involves our use of measures of access and continuity that were reported by clinics since we did not validate them as part of this study. Since this study cannot determine causality, there may have been unmeasured primary care clinic features that were related to access and continuity and ED visits. The estimates of access could be overestimated if we omitted significant clinic factors. While our study occurred during implementation of the PCMH/PACT model, we did not have data on whether study practices were fully functioning as patient-centered medical homes based on certification such as NCQA. We also did not have measures of whether PC-MHI teams were providing same-day access in practices, so we were unable to address the question of whether better access through PC-MHI could decrease ED visits for mental health conditions. Finally, the findings of this study may not be generalizable to primary care clinics outside of the VHA system since VHA is highly integrated with a national electronic medical record system.

### Conclusion

To reduce overcrowding in EDs and contribute to more efficient use of health care resources, improvements in primary care access can decrease ED visits that are for non-emergent and primary care treatable conditions. ED visits for mental health, however, were not sensitive to same-day access or continuity in primary care, so improving these primary care attributes alone may not significantly reduce mental health ED care without additional approaches such as better referral systems to and coordination with specialty mental health care providers.

## Supporting Information

S1 Table Standard Deviations Added to Table of Number of Emergency Department Visits Per 100 Primary Care Patients by Year and Type, N = 71,296. (DOCX)

S2 Table Standard Deviations Added to Table of Annual Rate of ED Visits by Patient and Clinic Characteristics FY2010-2012, N = 71,296. (DOCX)
