# Supplementary material for: The Relationship between Same-Day Access and Continuity in Primary Care and Emergency Department Visits
Source: PLoS One. 2015 Sep 2;10(9):e0135274. doi: 10.1371/journal.pone.0135274 (PMC4557991; doi:10.1371/journal.pone.0135274)
Supplement: S1 Table — (DOCX) [file pone.0135274.s001.docx]

**S1 Table. Standard Deviations Added to Table of Number of Emergency Department Visits Per 100 Primary Care Patients by Year and Type, N=71,296**

| **Type of ED visit** | **2010** | | **2011** | | **2012** | | **P-value from one way ANOVA** |
| --- | --- | --- | --- | --- | --- | --- | --- |
|  | **Mean** | **SD** | **Mean** | **SD** | **Mean** | **SD** |  |
| All-cause† | 55.5 | 178 | 55.7 | 172 | 58.1 | 184 | 0.0117 |
| Non-emergent | 10.2 | 44 | 10.6 | 43 | 11.3 | 45 | <0.0001 |
| Emergent |  |  |  |  |  |  |  |
| Primary care treatable | 11.8 | 50 | 11.4 | 44 | 11.8 | 47 | 0.2666 |
| ED care needed and preventable | 4.1 | 23 | 4.4 | 26 | 4.3 | 24 | 0.0355 |
| ED care needed and not preventable | 7.6 | 40 | 7.4 | 39 | 8.0 | 48 | 0.0149 |
| All mental health diagnoses | 3.5 | 36 | 3.4 | 35 | 3.1 | 35 | 0.0842 |
| Psychiatric | 2.2 | 24 | 2.1 | 21 | 1.7 | 20 | 0.0002 |
| Alcohol use | 1.1 | 23 | 1.2 | 24 | 1.2 | 24 | 0.6710 |
| Drug use | 0.2 | 5 | 0.2 | 6 | 0.2 | 5 | 0.5779 |

† All-cause ED visits included ED visits that were not categorized into one of the specific categories listed here.
