# Supplementary material for: The Relationship between Same-Day Access and Continuity in Primary Care and Emergency Department Visits
Source: PLoS One. 2015 Sep 2;10(9):e0135274. doi: 10.1371/journal.pone.0135274 (PMC4557991; doi:10.1371/journal.pone.0135274)
Supplement: S2 Table — (DOCX) [file pone.0135274.s002.docx]

**S2 Table. Standard Deviations Added to Table of Annual Rate of ED Visits by Patient and Clinic Characteristics FY2010-2012, N=71,296**

| **Patient and Clinic Factors** | **N (%)** | **Mean ED Visits/100 Patients per Year*** | **SD** | **P-value from one way ANOVA** |
| --- | --- | --- | --- | --- |
| ***Clinic factors*** |  |  |  |  |
| **Access: Percent of patients receiving same day appointment within 1 day** |  |  |  | <0.001 |
| <40% | 24530 (34) | 60.9 | 173 |  |
| 40%+ | 46766 (66) | 54.1 | 181 |  |
| **Provider Continuity: Ratio of outpatient visits to primary care provider** |  |  |  | <0.001 |
| <79% | 49095 (67) | 66.7 | 189 |  |
| 79%+ | 23993 (33) | 34.2 | 152 |  |
| **Clinic type** |  |  |  | <0.001 |
| VHA medical center-based clinic | 20563 (28) | 81.4 | 217 |  |
| Community-based outpatient clinic | 52525 (72) | 46.3 | 159 |  |
| **Metropolitan area** |  |  |  | <0.001 |
| No | 1925 (3) | 25.0 | 123 |  |
| Yes | 71163 (97) | 57.3 | 180 |  |
| **Distance to VHA medical center** |  |  |  | <0.001 |
| <16 miles | 40122 (55) | 71.2 | 197 |  |
| 16+ | 32966 (45) | 37.5 | 149 |  |
| ***Patient factors*** |  |  |  |  |
| **Age group** |  |  |  | <0.001 |
| <45 | 8982 (13) | 65.0 | 191 |  |
| 45-54 | 10414 (15) | 74.4 | 206 |  |
| 55-64 | 24122 (33) | 65.2 | 199 |  |
| 65+ | 27778 (39) | 39.4 | 153 |  |
| **Gender** |  |  |  | <0.001 |
| Female | 3920 (5) | 75.2 | 180 |  |
| Male | 67376 (95) | 55.3 | 178 |  |
| **Race/ Ethnicity** |  |  |  | <0.001 |
| White | 34953 (49) | 63.6 | 203 |  |
| Black | 10239 (14) | 77.8 | 191 |  |
| Hispanic | 9016 (13) | 61.5 | 174 |  |
| Other/Unknown | 17088 (24) | 25.9 | 129 |  |
| **Marital Status** |  |  |  | <0.001 |
| Married | 32995 (47) | 40.8 | 126 |  |
| Separated/Divorced/Widowed | 25372 (36) | 70.7 | 215 |  |
| Single Never married | 12657(18) | 69.0 | 209 |  |
| Unknown | 272 (<1) | 34.4 | 148 |  |
| **Homeless** |  |  |  | <0.001 |
| Yes | 3951 (5) | 143.6 | 353 |  |
| No | 67345 (95) | 51.3 | 161 |  |
| **Means Test** |  |  |  | <0.001 |
| Below Means Test, Not Service Connected | 25107 (35) | 70.2 | 222 |  |
| Service Connected | 30904 (43) | 58.1 | 164 |  |
| Above Means Test | 11103 (16) | 26.4 | 101 |  |
| Other Eligibility | 4182 (6) | 40.9 | 122 |  |
| **Service Connected Percent** |  |  |  | <0.001 |
| 0 | 41894 (59) | 55.0 | 187 |  |
| 1-50 | 16121 (23) | 50.0 | 144 |  |
| 51-100 | 13281 (18) | 68.9 | 187 |  |
| **Charlson Index** |  |  |  | <0.001 |
| 0 | 34706 (49) | 37.2 | 131 |  |
| 1+ | 36590 (51) | 74.7 | 212 |  |
| **Chronic conditions** |  |  |  | <0.001 |
| CHF | 2890 (4) | 155.5 | 301 |  |
| COPD | 6007 (8) | 115.8 | 296 |  |
| Asthma | 3101 (4) | 113.5 | 319 |  |
| Ischemic heart disease | 10382 (15) | 86.7 | 256 |  |
| Diabetes | 19894 (28) | 69.5 | 193 |  |
| Hypertension | 42285 (59) | 64.8 | 196 |  |
| Drug use | 3440 (5) | 197.7 | 439 |  |
| Alcohol | 4533 (6) | 156.1 | 365 |  |
| Depression | 15085 (21) | 100.4 | 274 |  |
| Other Psychiatric disorders | 22955 (32) | 91.0 | 252 |  |
| PTSD | 10454 (15) | 88.1 | 240 |  |
| **Number of primary care visits** |  |  |  | <0.001 |
| 0-2 | 32832 (46) | 22.7 | 89 |  |
| 3+ | 38464 (54) | 85.2 | 224 |  |
| **Number of telephone visits** |  |  |  | <0.001 |
| 0 | 51820 (73) | 37.6 | 138 |  |
| 1+ | 19476 (27) | 106.4 | 249 |  |

*Annual rate of ED visits per 100 patients is the mean number of ED visits per patient per year across all study years multiplied by 100.
